# Water adsorption on TiO_2_ surfaces probed by soft X-ray spectroscopies: bulk materials vs. isolated nanoparticles

**DOI:** 10.1038/srep15088

**Published:** 2015-10-14

**Authors:** Safia Benkoula, Olivier Sublemontier, Minna Patanen, Christophe Nicolas, Fausto Sirotti, Ahmed Naitabdi, François Gaie-Levrel, Egill Antonsson, Damien Aureau, François-Xavier Ouf, Shin-Ichi Wada, Arnaud Etcheberry, Kiyoshi Ueda, Catalin Miron

**Affiliations:** 1 Synchrotron SOLEIL, L’Orme des Merisiers, Saint-Aubin, BP 48, 91192 Gif-sur-Yvette Cedex, France; 2 CEA/IRAMIS/NIMBE/Laboratoire Edifices Nanométriques, CEA Saclay, 91191 Gif-sur-Yvette, France; 3 Sorbonne Université UPMC, Univ Paris 6, UMR 7614, Laboratoire de Chimie Physique Matière et Rayonnement, 11 rue Pierre et Marie Curie, 75005 Paris, France; 4 Laboratoire national de métrologie et d’essais, département “métrologie des gaz et des aérosols”, 1 rue Gaston Boissier, 75724 Paris Cedex 15; 5 Institut Lavoisier de Versailles, Université Versailles-St Quentin, UMR CNRS 8180, 78035 Versailles, France; 6 Institut de Radioprotection et de Sûreté Nucléaire (IRSN), PSN-RES, SCA, LPMA, 91192 Gif-Sur-Yvette, France; 7 Graduate School of Science, Hiroshima University, Higashi-Hiroshima 739-8526, Japan; 8 IMRAM, Tohoku University, Sendai 980-8577, Japan; 9 Extreme Light Infrastructure - Nuclear Physics (ELI-NP), “Horia Hulubei” National Institute for Physics and Nuclear Engineering, 30 Reactorului Street, RO-077125 Măgurele, Jud. Ilfov, Romania

## Abstract

We describe an experimental method to probe the adsorption of water at the surface of isolated, substrate-free TiO_2_ nanoparticles (NPs) based on soft X-ray spectroscopy in the gas phase using synchrotron radiation. To understand the interfacial properties between water and TiO_2_ surface, a water shell was adsorbed at the surface of TiO_2_ NPs. We used two different ways to control the hydration level of the NPs: in the first scheme, initially solvated NPs were dried and in the second one, dry NPs generated thanks to a commercial aerosol generator were exposed to water vapor. XPS was used to identify the signature of the water layer shell on the surface of the free TiO_2_ NPs and made it possible to follow the evolution of their hydration state. The results obtained allow the establishment of a qualitative determination of isolated NPs’ surface states, as well as to unravel water adsorption mechanisms. This method appears to be a unique approach to investigate the interface between an isolated nano-object and a solvent over-layer, paving the way towards new investigation methods in heterogeneous catalysis on nanomaterials.

Titanium dioxide (TiO_2_) is undoubtedly one of the most studied materials owing to its technological relevance to various fields, such as photonics, electronic devices, self-cleaning materials and photocatalysis^1^,^2^,^3^,^4^. Considerable research effort has been devoted to the understanding of the link between the surface properties of TiO_2_ and water adsorption mechanisms on its surface^5^,^6^,^7^,^8^. These mechanisms are known to be essential in photocatalytic processes, affecting for instance charge recombination rates^9^,^10^. The water - TiO_2_ interface is thus of crucial importance, and the proper control of the surface properties of TiO_2_ appears to be even more crucial, as soon as we reach the nanometer scale where the surface-to-bulk ratio is considerably larger than in the infinite solid. Several studies, based on techniques such as high resolution scanning tunneling microscopy (HRSTM)^7^,^11^,^12^ or X-ray Photoelectron Spectroscopy (XPS)^13^,^14^, have been reported in the literature and aimed at understanding and controlling TiO_2_ nanoparticles (NPs)’ surface properties. However, despite the wide interest devoted to this subject, the photocatalytic activity of TiO_2_ NPs and its correlation with water adsorption is still a matter of debate both theoretically and experimentally because of the complex interplay between the surface structure at the atomic scale and the nature of the adsorption mechanisms^5^,^7^,^15^,^16^,^17^. The degree of complexity is also enhanced by the fact that the NPs are usually deposited on a substrate, resulting in sample modifications during the deposition process itself, interactions between the substrate and nanosystem under study, and sample charging effects. Identifying the key factors influencing the adsorption mechanisms and mastering the degree of hydration of TiO_2_ NPs are important challenges for photocatalysis. Here we address the water adsorption problem on the surface of isolated TiO_2_ NPs using a novel experimental technique, which has recently proved its efficiency in the characterization of isolated nano-objects^18^,^19^,^20^. Our approach consists in using synchrotron radiation (SR) based soft X-ray electron spectroscopy to analyse the properties of a collimated beam of differently hydrated NPs generated and focused to the interaction region with the SR by an Aerodynamic Lens System (ADLS). This experimental approach offers the opportunity of avoiding any interaction between the sample and a substrate, thus giving access to the sole, intrinsic information about the NP surface. Several questions have been addressed to evaluate the feasibility of controlling the hydration state of freestanding TiO_2_ NPs and to achieve insight into the factors which can influence the water adsorption mechanisms on isolated TiO_2_ NPs in the gas phase.

## Results

**Figure 1:**
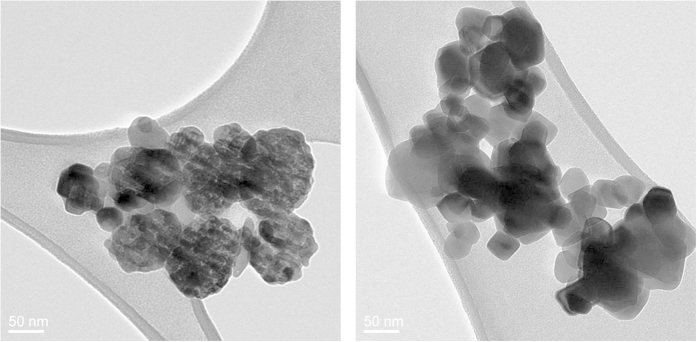
TEM images of commercial TiO_2_ nanopowder mixture of rutile and anatase from *Sigma Aldrich* (**a**) and pure anatase from *MK Impex* Corp. (**b**).

A variety of studies was preliminary conducted in view of the structural characterization of the commercial TiO_2_ nanopowder. The TEM images obtained for the TiO_2_ nanoparticles reveal an important size dispersion of the nanometer grains (Fig. 1), ranging from 20 nm to 120 nm. The sample from Sigma Aldrich, made of a mixture of the two prevalent crystalline phases of TiO_2_, namely rutile and anatase (Fig. 1a) shows clearly the presence of two distinguishable morphotypes on the grain structure – a faceted and a cluster-like structure – which can be attributed to the two different crystalline phases present in the sample. This assumption is supported by the absence of such an inhomogeneity on the micrograph performed on a commercial sample of pure anatase TiO_2_ nanopowder (MK Impex Corp.) (Fig. 1b). A systematic TEM study of different samples additionally shows that the amount of anatase and rutile phases present in the mixture is not equivalent. The X-ray diffraction (XRD) patterns of all samples (not shown here) evidence a predominant anatase phase in the mixture with large anatase (101) and (200) peaks, along with a weak rutile (110) peak. Using Spurr and Myers formula^21^, the fraction of anatase in the commercial nanopowder was thus evaluated to be 0.8. Some DFT calculations and experimental studies have shown that the water sorption mechanisms are dependent on the crystalline structure^22^, as well as on the orientation of nanocrystals^5^,^23^,^24^. However, as our gas-phase experiment results to an averaging of the contributions from all crystal orientations, the difference between anatase and rutile becomes meaningless. The mixture sample of 100 nm was thus chosen to be close to a “realistic” sample commonly used in applications. Even if the anatase-rutile NPs mixture of 25 nm (P25) is the most used in commercial photocatalysis systems, it is poorly focused with our ADLS. Consequently, the choice of the average size of nanopowder sample (100 nm) was mainly guided by our aerodynamic focusing efficiency requirements.

**Figure 2:**
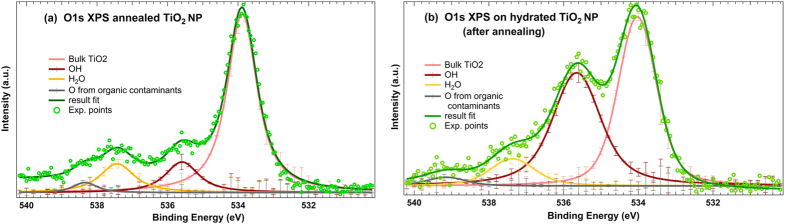
Experimental fit performed on O 1s XPS spectra obtained for annealed TiO_2_ nanopowder before hydration (**a**) and after hydration at 90% of RH (**b**) using synchrotron radiation.

Soft x-ray photoelectron spectroscopy was initially performed on dry TiO_2_ NPs sprayed out by the nanopowder aerosoliser and then hydrated thanks to the setup described in the *Methods* section. In order to ensure the dryness of the particles prior to aerosolisation, an annealing process has been performed, similarly to the protocols used in bulk surface science. For annealing, the nanopowder has been kept in a vacuum oven at 150 °C under N_2_ atmosphere during 24 h. The temperature has been chosen deliberately low in order to avoid any crystalline phase transition, according to previous observations^25^,^26^. The effect of annealing on the O 1s XPS spectra can be seen in Supplementary Figure S1 online. The annealed nanopowder was then transferred in the aerosoliser chamber before being sprayed through the ADLS. In these conditions, the exposure of the nanopowder to the ambient moisture is strictly limited; however a brief exposure during the transfer to the aerosoliser chamber remains possible. Figure 2 displays O 1s XPS core-level spectra obtained for annealed nanopowder before hydration (a) and during hydration (b) by water evaporation as described in the *Methods* section. The incident photon energy used to record the O 1s spectra was 630 eV, leading to an inelastic mean free path of about 0.6 nm in TiO_2_, as determined by using Seah’s equation for inorganic compounds^27^. The experimental resolution (originating from the convolution of the monochromator bandwidth and of the electron spectrometer resolution) was about 960 meV, resulting in a total FWHM in the range 1.2–1.5 eV for each component (see Supplementary Table S2 online). It is worth stressing that NPs samples present a non-cleaned, non-oriented surfaces which are all measured at the same time, and each of the spectral lines represents an ensemble of atomic arrangements in the NPs with slightly different chemical environments (leading to slightly different binding energies). Also, on top of the instrumental and lifetime broadenings, linewidths are affected by phonon and final state vibrational broadenings (FSVB). In case of absorbed species the FSVB can lead to broader photolines than their gas phase counterparts due to the formation of new vibrational modes^28^,^29^. For example several OH-vibrational modes having frequencies up to 0.5 eV have been reported on hydroxylated TiO_2_ surfaces^30^.

A systematic energy calibration has been performed, by recording the Ar 2p photoemission lines in the gas phase. A Voigt profile was used to fit the data, and the background was assumed to have a Shirley-type shape^31^,^32^. In order to achieve a reasonable fit of the broad structure originating from O 1s photoemission, four symmetric peaks with their experimental linewidths as fixed parameter were used. For information, the error bars representing the standard deviation have been reported for the individual fitting components. The relative energy positions of the components have also been kept constant (within the error bars), except for the higher binding energy (BE) peak whose position is shifted as discussed below.

Hence, after Shirley background subtraction and deconvolution, the spectrum gives rise to four components as shown in Fig. 2. The positions of each component used to fit the spectra can be found in Supplementary Table S3 online. The main peak at 533.8 ± 0.2 eV BE is interpreted as originating from bulk oxygen atoms in the TiO_2_ NPs lattice, and the component at 535.6 ± 0.2 eV BE is linked to the adsorption of water on the TiO_2_ surface, mainly as hydroxylated chemisorbed species, which is in agreement with several previous bulk studies^14^,^25^,^31^,^33^,^34^ and confirmed by the fact that before hydration, this component is substantially reduced (Fig. 2a) and increases as a function of the water temperature during hydration (Fig. 2b). Let us point out that even without hydration, a small residual component attributed to the position of OH species still remains in the spectrum (a). This can be linked to the spectroscopic signature of the two-fold coordinated O-bridging, which has been already evidenced by Bullock *et al.* and other groups^32^,^35^. However, a small hydration due to ambient moisture is not totally excluded to explain the presence of this peak.

Two different OH groups have been distinguished in the literature of hydrated TiO_2_ surfaces^32^,^35^,^36^: OH-groups bond to 5-coordinated Ti^4+^ cations forming Ti-OH basic groups, and acidic OH-groups linked to the bridging oxygens, here called O_*br*_H. Sham and Lazarus reported already in 1979 a study of hydrated rutile (001) surface, where they observed these chemisorbed acidic and basic groups as well as physisorbed components in the O 1s XPS^36^. Evaluating from the spectra they present, the BE shifts from the bulk O 1s component are approximately +1.6 eV and +2.6 eV for O_*br*_H and Ti-OH components, respectively. Perron *et al.* reported a binding energy shift of +1.3 eV for O-bridging component, and +2.5 eV for Ti-OH. Unfortunately, we cannot resolve these peaks, and only one broad component (FWHM = 1.5 eV) is fitted with a binding energy shift of +1.8 eV compared to the bulk O, representing non-hydrated O-bridging, O_*br*_H, and Ti-OH.

To fit the experimental data, two more components have to be added: an intermediate peak at 537.4 ± 0.2 eV BE which results from the adsorption of molecular water in the upper layer, as previously shown by Sham and Lazarus^36^, H. Perron *et al.*^32^ and other groups^37^, and a higher BE component whose position relative to the bulk component seems to fluctuate. This peak can be related to oxygen from organic contaminants at the surface of TiO_2_ NPs resulting either from the annealing process^38^ or from air exposure. The energy location of the carbon contamination signature is known to depend on the nature of the adsorbate and to vary for different organic contaminants as a function of the chemical partner of the carbon (e.g. oxygen or hydrogen)^39^. This chemical shift can thus be assigned to the different neighboring of the organic adsorbent species before (a) and during (b) hydration. It is important to stress that the energy scale of all spectra is referenced to the vacuum level, due to the gas phase configuration. Consequently, a systematic shift varying between 3.5 eV and 4 eV of the whole spectrum is to be considered to compare with the data from the literature dealing with deposited NPs, which corresponds to the TiO_2_ work function.

**Figure 3:**
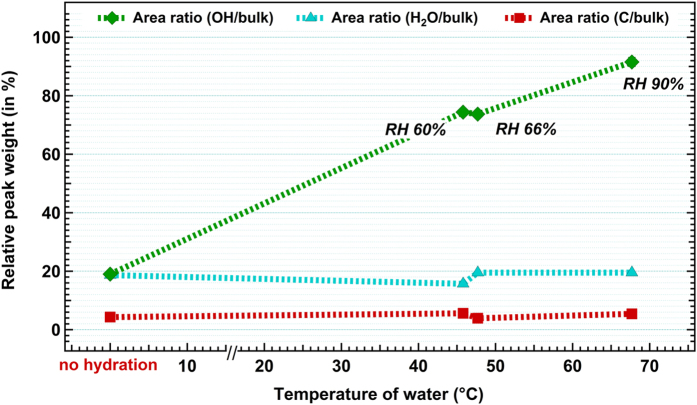
Area ratio of each component of the O 1s spectra relative to the bulk component after peak fitting. The first point corresponds to the spectrum recorded before hydration. Error bars are within the size of markers.

Evaluations of the peak areas relative to the bulk component for different hydration levels are shown in Fig. 3. The relative humidity (RH) measured for each step of water-heating temperature is also reported. The curves reveal that the peak weight at 535.6 eV related to the OH species adsorbed is strongly dependent on the RH, whereas the H_2_O peak seems to stay constant whatever the hydration level is. It has been shown that molecular physisorbed water is easily desorbed under ultrahigh vacuum conditions^32^,^37^, and hence cannot be directly linked to the hydration level. The higher BE peak is also independent of the state of hydration which supports the assignment to oxygen atoms from organic contaminants, whose signature is also confirmed by the slightly grey color observed on our nanopowder samples after annealing.

To validate this interpretation of the spectra, additional XPS measurements have been performed for chemical specification of our sample with a monochromatized Al K*α* source on TiO_2_ NPs deposited on a substrate, as described in the *Methods* section.

**Figure 4:**
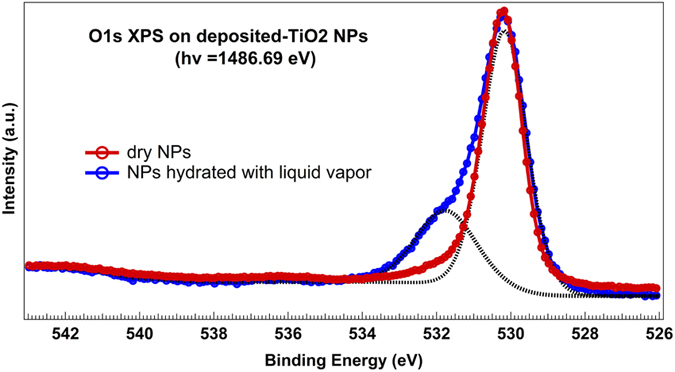
O 1s XPS spectra of dry (*red*) and hydrated (*blue*) deposited TiO_2_ NPs, obtained with an Al K*α* source. The dotted line presents the fit result of the blue spectrum.

Figure 4 displays the O 1s XPS spectra obtained with the latter setup. Charge effects are compensated thanks to a flood gun correction and a systematic calibration by recording C1s position. Fitting has been performed with the commercial *Thermo scientific Avantage* software, using a Voigt line shape. The bulk oxygen peak arises at 530.2 eV BE in perfect agreement with the position observed by Hugenschmidt *et al.*^40^. The BE shift of the overall spectrum with regards to Fig. 2 - corresponding to gas phase configuration - enables to extract a work function equal to 3.6 eV.

As was the case for the isolated nanoparticles, a second component arises at 1.6 eV higher BE upon hydration (blue spectrum) which is fully consistent with the peak position attributed to OH chemisorbed species in Fig. 2, observed at +1.8 eV from the bulk O peak. This peak was verified to be independent from any C-contamination, by recording C 1s XPS spectra before and after hydration. It has to be stressed that due to a lower surface sensitivity in the latter conditions, the NPs were saturated with liquid water in order to get a signal from surface-adsorbed water, resulting in a higher coverage in this case. It might also be noted that no other component is observed at higher energy, contrary to the spectra shown in Fig. 2. This confirms the assignment of the peak at 537.4 eV BE in Fig. 2 to physisorbed molecular water in the upper layer, which tends to be more easily desorbed under the present higher vacuum conditions. It explains why this peak has been evidenced only at low temperature^41^ or by tilting the sample^32^.

**Figure 5:**
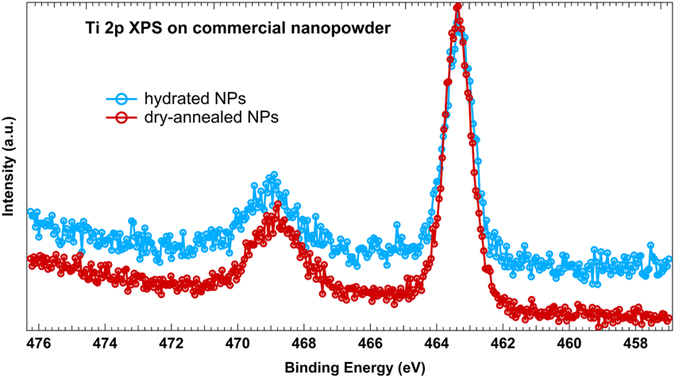
Ti 2p XPS spectra obtained on annealed NPs without hydration (*red spectrum*) and during hydration with water evaporation at 68 °C (*blue spectrum*) using synchrotron radiation. The incident photon energy was 560 eV.

Ti 2p XPS spectra have also been recorded on freestanding TiO_2_ NPs, before and after hydration of previously annealed TiO_2_ NPs. The Ti 2p core-level spectra (Fig. 5) do not reveal any obvious signature of water adsorption. However, some studies report the presence of a shoulder at the lower binding energy side of the Ti 2p doublet^31^,^33^,^42^,^43^ attributed to reduced species (Ti^3+^ and Ti^2+^) in non-stoichiometric defective TiO_2_ films. This asymmetry is observed to be quenched after subsequent water exposure^33^,^43^, resulting in a “healing” of surface defects as shown by Wang *et al.*^33^. The absence of such an asymmetry in our situation and the high similarity between the two spectra in Fig. 5 reveals that these Ti 3d states are absent or below the detection threshold in our NPs and suggests that they do not take part in the adsorption path. Thus, we can conclude that the Ti-OH are not most likely dominating species on the surface of NPs and in the O 1s spectra the strong increase of OH-group contribution is mostly due to the O_*br*_H-groups.

**Figure 6:**
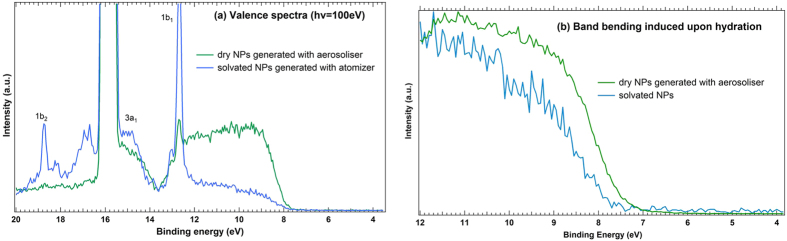
(**a**) Valence spectra obtained with a suspension of solvated NPs sprayed out with an atomizer (*blue*) and on “as-received” dry NPs sprayed out with an aerosoliser (*green*) at 100 eV photon energy. (**b**) Comparison of the edge of the valence band (4–12 eV) for “as-received” dry NPs (*green*) and hydrated NPs (*blue*). The spectra have been normalized relative to the top of the valence band.

However, a clear evidence of water adsorption can be seen in the valence spectra. Indeed, Fig. 6a depicts the valence spectra obtained on solvated TiO_2_ NPs sprayed out by atomization (blue spectrum) and on an “as-received” nanopowder sprayed out with the commercial nanoaerosoliser (green spectrum). The peak at 15.9 eV BE corresponds to Ar 3p valence states, and has been deliberately kept for calibration purposes. The spectrum corresponding to solvated TiO_2_ NPs gives rise to three molecular states of water in the valence band region, labelled as 1b_1_, 3a_1_ and 1b_2_ – as previously described by M.A. Henderson^44^ – which are absent or strongly attenuated in the spectrum of dry NPs. Water peak assignment was based on Kimura’s *et al.*^45^ valence states study. The presence of molecular water states in the valence band region (blue spectrum) is assigned to the atomization procedure of NPs in water suspension. However, it is difficult to distinguish the signal of adsorbed surface water from gas phase water also present in the interaction region, because the binding energy shift between them is very small in the valence band region. To better illustrate water adsorption in the valence band region, the spectra have been normalized relative to the top of the valence band (Fig. 6b) and energy calibration was achieved using the Ar 3p photoline. A difference between dry and solvated NPs cases is evidenced: the band bending of the valence edge accompanying the hydrated state and the shift towards higher BE is in full agreement with the observation made by Kurtz *et al.*^46^ on bulk hydrated TiO_2_ surfaces. The same behaviour was highlighted with DFT calculations^47^ and was attributed to a solvation of TiO_2_ surface states due to water adsorption.

## Discussion

Considerable effort has been dedicated to unravel the reactivity of water on TiO_2_ surfaces. However, the literature suffers from insufficient information regarding the nanoparticles’ case. Even on the well-known bulk situation, several factors have been shown to influence the water sorption scheme. Some studies have argued for a coverage-dependency on the adsorption mechanism, with a two-step process in which a low coverage dissociative adsorption is followed by a molecular adsorption at higher coverage^16^,^43^. Another point of view defends influence of the sample temperature on the adsorption scheme^46^. However, a large consensus seems to be achieved for a molecular adsorption on defect-free surfaces, whereas dissociation occurs only on defective surfaces^24^ especially at O-vacancies (O_*vac*_)^8^,^11^,^25^. Walle *et al.*^41^ have recently shown that dissociation can also take place on vacancy-free surfaces, resulting in a mixed dissociative and molecular water adsorption qualified as a “pseudodissociated state” at monolayer coverage, where the temperature of the substrate plays a crucial role in the experimental observation of such a mixed state.

**Figure 7:**
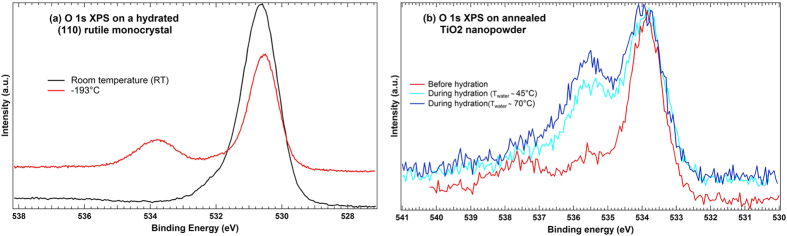
O 1s XPS spectra recorded on bulk rutile (110) monocrystal at the TEMPO beamline (**a**) and on our commercial nanopowder (mixture of rutile and anatase) at the PLEIADES beamline (**b**).

SR-based XPS of a rutile (110) single crystal was recorded to follow the hydration signature of a bulk solid in ultra-high vacuum conditions for comparison with the isolated NPs experiment using the same photon energy (Fig. 7a).

In good agreement with the experiments of Walle *et al.*^41^ these results show that (*1*) *no trace of molecular water can be visible at room temperature* (*RT*) *whereas it is clearly evidenced at −193 °C through the structure around 534 eV,* (*2*) *a small shoulder appears at 532.2 eV at both temperatures, and seems to be related to OH species chemisorbed at O*_*vac*_. Contrary to Walle’s experiment, the present study is made on a defective surface with O_*vac*_ – as observed with the STM characterization performed on the rutile (110) monocrystal (*not shown here*) – supporting the view that in this case, OH species are preferentially adsorbed at O_*vac*_ sites.

For comparison, the data obtained with our isolated nanoparticles (mixture of rutile/anatase) are shown in Fig. 7b on the annealed nanopowder progressively hydrated. As previously discussed, the spectrum corresponding to dry-annealed NPs, as well as the spectra associated to a controlled-hydration, display four components with a clear signature of OH and H_2_O – as supported by the fit – the whole hydration procedure being made at RT. Moreover, still in contradiction with the bulk situation, the hydration is accompanied by a strong increase of the OH component - with a weak effect on the H_2_O peak - giving rise to the second peak at 535.6 eV, which corresponds to the small shoulder observed on the (110) surface (Fig. 7a).

In a classical paper by Sham and Lazarus^36^, chemisorbed and physisorbed water on a freshly introduced ambient sample of TiO_2_ (001) surface were observed. When the sample was allowed to stay in UHV vacuum conditions for a week, the high energy side of the O 1s spectrum was substantially decreased, indicating that these features corresponded to physisorbed water. A similar effect can be observed in our spectrum of the (110) surface (Fig. 7a) evidencing the presence of physisorbed water: when the temperature is decreased, the residual moisture of the experimental chamber condenses on the TiO_2_ surface and the physisorbed molecular water peak becomes evident. Additionally, in their study Sham and Lazarus mechanically scraped the surface before exposing it to water, thus creating a lot of surface defects. To record the O 1s XPS they used Mg K*α* radiation, resulting to higher kinetic energies and thus longer escape depth of the photoelectrons, and in order to be more sensitive to the surface, the sample was tilted which clearly enhanced the intensity of the hydroxylated peaks. The effect is very similar to what can be seen in the evolving hydration process of NPs (Fig. 7b). However, compared to our results obtained on a rutile (110) monocrystal and to the (001) surface studied by Sham and Lazarus, the NPs data show stronger increase of the OH-species.

The first explanation for this difference in intensity of the OH component can be attributed to the coverage, which is higher in the case of isolated TiO_2_ NPs than with a well-controlled surface, the hydration process being hardly quantifiable in our gas phase environment. This coverage dependency is moreover confirmed by the presence of a molecular H_2_O peak in Fig. 7b, which can be observed only at low temperature on the experimental data of the (110) surface (Fig. 7a) or on Walle’s study^41^. Decreasing the temperature might result in adsorption of residual water on the surface of TiO_2_ and prevent from complete desorption of adsorbed species as shown in other studies^24^,^37^,^41^. This supports the idea that at higher coverage the adsorption can also occur molecularly on the OH sublayer acting as “anchor sites” for H_2_O molecules, as reported by Yamamoto *et al.*^43^ for the TiO_2_ (110) surface. Indeed, the presence of the molecular H_2_O component was also experimentally evidenced by H. Perron and co-workers^32^ on a (110) rutile surface at RT where the hydration process was performed during 24 h, resulting in a presumed higher hydration amount than in Fig. 7a, and more comparable with our isolated NPs hydration conditions.

Let us point out that the relative coverage is moreover maximized at the nanometer scale, where the surface-to-bulk ratio is larger, resulting in an exaltation of the water-peaks, which become almost comparable to the bulk component as the water layer thickness increases.

**Figure 8:**
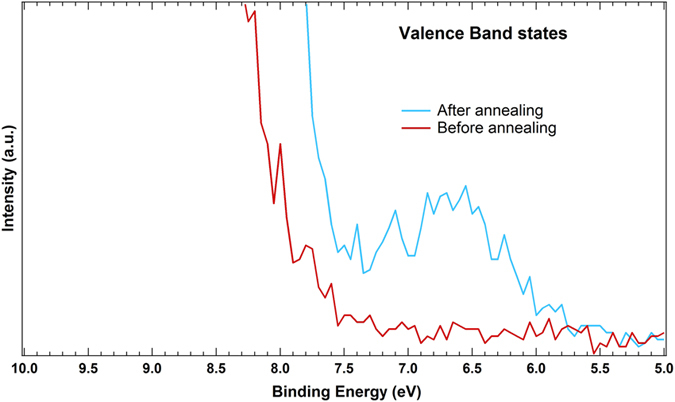
Band gap states induced by the annealing process.

A clear indication for the occurrence of a dissociative adsorption mechanism is hence visible through our NP spectra via the appearance of a RH-dependent OH component, and this is consistent with the fact that our commercial nanopowder sample might contain O_*vac*_ at the NPs’ surface, especially after the low-temperature annealing process carried out to dehydrate the nanopowder. The presence of induced-surface defects is confirmed by the appearance of a shoulder in the valence gap region for the annealed nanopowder samples (Fig. 8), which was absent from the non-annealed sample.

These valence gap states are usually attributed either to O_*vac*_ or Ti interstitials^37^. However Yim *et al.*^48^ have shown that these states are mainly resulting from O_*vac*_ rather than Ti interstitials, which is consistent with the fact that no Ti^3+^ component was observed in our Ti 2p core-level spectrum. A DFT study has also attributed this peak at +1 eV above the VB of TiO_2_ to a “poorly solvated” configuration of OH species^49^.

**Figure 9:**
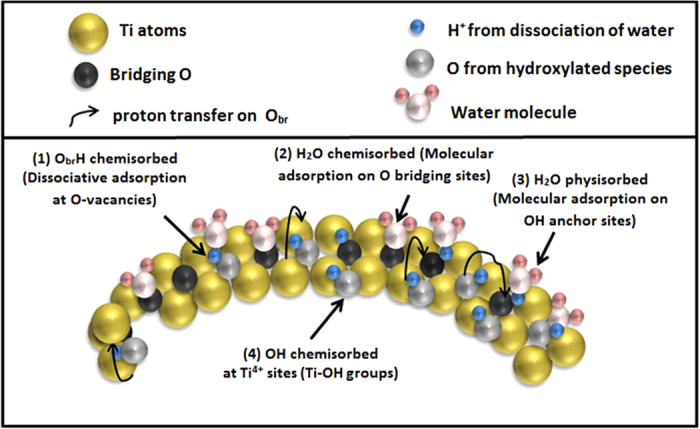
Schematic diagram of the proposed adsorption mechanisms at the surface of a TiO_2_ nanoparticle. The four main mechanisms which can occur on a TiO_2_ nanoparticle surface are displayed on the Figure.

Based on this XPS study, Fig. 9 summarizes the main adsorption schemes which can occur at the surface of a TiO_2_ NPs and evidenced through XPS spectra measurements. The dissociative mechanism (*1*) at O-vacancies (grey O atoms) have been shown to be dominating, resulting in a highly hydroxylated surface. A proton transfer on a neighbouring O-site can subsequently occur (black O atoms), resulting in a OH weight which is twice the density of defects, as already shown^11^,^46^. Related to this “chemisorption path” a molecular H_2_O adsorption which could contribute to the peak arising under hydration (Fig. 7b) is not totally excluded (*2*). Increasing the coverage, the H_2_O physisorption takes place in the upper layers (*3*), which is enhanced by the presence of underlying OH sites. Another dissociative mechanism (*4*) via 5-coordinated Ti atoms adds also the amount of O_*br*_H species when the proton is transferred from Ti to the neighboring O-bridging atom (schematically represented by black arrows). This pathway is concluded to be a smaller contribution compared to the mechanism (*1*), based on the fact that the Ti 2p XPS spectra remained unchanged during the hydration process. Also the fitted OH-related peak in the hydrated nanoparticle XPS shows a BE shift closer to the O_*br*_H value from literature, but one has to be careful when comparing the importance of these methods based on O 1s XPS since both mechanisms (*1*) and (*4*) contribute to the O_*br*_H species.

For the first time, we have experimentally demonstrated the presence of water at the surface of freestanding TiO_2_ NPs. To achieve this, we used aerodynamic focusing and SR-based X-ray photoelectron spectroscopy. More importantly, we have shown that it is possible to control the amount of water adsorbed at the NPs’ surface and to unravel the adsorption mechanism. A comparison with the bulk case showed that the water signature is exalted in the O 1s XPS spectra through the OH component, attributed to a higher coverage and the higher surface-to-bulk ratio, enhancing the weight of the water components. This result is relevant and of high importance to the photocatalysis research, taking into account that the dissociation of water is favored at the surface of TiO_2_ NPs. The high surface sensitivity obtained in our experimental conditions have proved to be crucial to disentangle the role of the surface state into the adsorption mechanism. Finally, consistent with previous studies on bulk TiO_2_ surfaces, we have observed a clear evidence for a mixed dissociative and molecular adsorption mechanisms, explained by the high coverage obtained in our experimental hydration conditions for nanomaterials.

## Methods

### SR based soft X-ray electron spectroscopy measurements

The experiments were carried out at the French national SR laboratory SOLEIL (Saint-Aubin, France) at the ultrahigh resolution soft X-ray PLEIADES beamline (9–1000 eV), where soft X-rays with any kind of polarization can be generated using an Apple II – type permanent magnet HU80 (80 mm period) undulator starting from 60 eV. PLEIADES is dedicated to the spectroscopic studies of isolated species ranging from atoms^50^, ions^51^ and molecules^52^,^53^,^54^,^55^ to proteins^56^,^57^, clusters^58^ and nanoparticles^20^,^59^. The photoelectron spectra were recorded with a commercial VG-Scienta R4000 spectrometer based on a hemispherical electron analyzer whose detection axis is perpendicular to the propagation direction of the SR. The pass energy and entrance slit are selected according to the experimental resolution targeted for each measurement and the polarization vector of the linearly polarized SR was chosen to be parallel to the electron detection axis.

The basic idea of the experiment is to create a collimated beam of isolated TiO_2_ NPs in interaction with SR under high vacuum conditions. Briefly, a flow of nanoparticles is sprayed out in the aerosol phase using a carrier gas (usually Ar or N_2_) and the resulting solid aerosol is focused thanks to an ADLS and injected through a 2 mm skimmer into the high vacuum chamber of the photoelectron spectrometer setup where the pressure is kept around 2 × 10^−6^ mbar during the experiment. The principle of the ADLS was widely described by Zhang *et al.*^60^ or McMurry *et al.*^61^,^62^,^63^ and its relevance to the study of isolated nanospecies has already been demonstrated at the PLEIADES beamline^20^,^59^,^64^. Complementary measurements of single crystal TiO_2_ have been performed at the TEMPO beamline at the French national SR facility SOLEIL (A. Naitabdi *et al.*). TEMPO is dedicated to the spectroscopic studies of condensed matter in the soft X-ray region (50–1500 eV), supplying a good complementarity to the gas-phase measurements performed at PLEIADES. The comparison is discussed in section “Discussion”.

### Hydration of the nanoparticles beam

To study water/TiO_2_ surface interaction and to control the hydration level of the TiO_2_ NPs, two different experimental methods have been used. The first method was based on the drying of solvated nanoparticles. Commercial TiO_2_ NPs (Sigma Aldrich, mixture rutile and anatase, diameter <100 nm) are initially kept in a distilled water suspension at a concentration of 5 g/L, and droplets are generated by atomization of the liquid suspension using a commercial atomizer setup (*Atomizer model 3076, TSI Inc.*). The droplets are then dried through a silica gel diffusion dryer and a tube furnace (*Vecstar*) before being transmitted into the ADLS system. The second method was based on the hydration of a dry particles beam generated with a commercial aerosoliser (*Naneum Aerosolizer PA100S*). The nanopowder aerosol is generated by concentrating high velocity vibrating jets of Ar under high pressure (6 bars) at the powder surface inside a vortex shaker aerosolisation chamber. Nitrogen gas was flown through a heated bubbler filled with deionized-water to hydrate the flow of NPs before the injection in the ADLS. The moisture level was controlled by varying water temperature and the flow rate of N_2_. Water temperature was measured thanks to a type-K thermocouple and the humidity level was measured *in-situ* with a commercial humidity sensor (*Sensirion, kit EK-H5*).

### Classical XPS measurements

A better characterization of our nanopowder has been achieved by additional XPS measurements carried out at the French laboratory “Institut Lavoisier de Versailles”, on deposited TiO_2_ NPs, using the same sample which served for the SR studies. The spectra were collected using an XPS apparatus (*Thermo scientific*) with a monochromatized Al K*α* source, and the pressure in the analysis chamber was kept around 2 × 10^−8^ mbar.

## Additional Information

**How to cite this article**: Benkoula, S. *et al.* Water adsorption on TiO_2_ surfaces probed by soft X-ray spectroscopies: bulk materials vs. isolated nanoparticles. *Sci. Rep.* **5**, 15088; doi: 10.1038/srep15088 (2015).

## Supplementary Material

Supplementary Information

## References

[b1] Simon P. *et al.* N-Doped Titanium Monoxide Nanoparticles with TiO Rock-Salt Structure, Low Energy Band Gap, and Visible Light Activity. Chem. Mater. 22, 3704–3711 (2010).

[b2] Scanlon D. O. *et al.* Band alignment of rutile and anatase TiO_2_. Nature Mater. 12, 798–801 (2013). 23832124 10.1038/nmat3697

[b3] Yola M. L., Eren T. & Atar N. A novel efficient photocatalyst based on TiO_2_ nanoparticles involved boron enrichment waste for photocatalytic degradation of atrazine. Chem. Eng. J. 250, 288–294 (2014).

[b4] Reddy K. M., Manorama S. V. & Reddy A. R. Bandgap studies on anatase titanium dioxide nanoparticles. Mater. Chem. Phys. 78, 239–245 (2003).

[b5] Diebold U. The surface science of titanium dioxide. Surf. Sci. Rep. 48, 53–229 (2003).

[b6] Borodin A. & Reichling M. Characterizing TiO_2_(110) surface states by their work function. Phys. Chem. Chem. Phys. 13, 15442–15447 (2011). 21779605 10.1039/c0cp02835e

[b7] Matthiesen J. *et al.* Formation and Diffusion of Water Dimers on Rutile TiO_2_(110). Phys. Rev. Lett. 102, 226101 (2009). 19658879 10.1103/PhysRevLett.102.226101

[b8] Aschauer U. *et al.* Influence of subsurface defects on the surface reactivity of TiO_2_: Water on anatase (101). J. Phys. Chem. C 114, 1278–1284 (2010).

[b9] Dimitrijevic N. M. *et al.* Role of Water and Carbonates in Photocatalytic Transformation of CO_2_ to CH_4_ on Titania. J. Am. Chem. Soc. 133, 3964–3971 (2011). 21348527 10.1021/ja108791u

[b10] Tang J., Durrant J. R. & Klug D. R. Mechanism of Photocatalytic Water Splitting in TiO_2_. Reaction of Water with Photoholes, Importance of Charge Carrier Dynamics, and Evidence for Four-Hole Chemistry. J. Am. Chem. Soc. 130, 13885–13891 (2008). 18817387 10.1021/ja8034637

[b11] Wendt S. *et al.* Oxygen vacancies on TiO_2_(110) and their interaction with H_2_O and O_2_: A combined high-resolution STM and DFT study. Surf. Sci. 598, 226–245 (2005).

[b12] Du Y. *et al.* Water Interactions with Terminal Hydroxyls on TiO_2_ (110). J. Phys. Chem. C 114, 17080–17084 (2010).

[b13] Chen X. & Burda C. The electronic origin of the visible-light absorption properties of C-, N- and S-doped TiO_2_ nanomaterials. J. Am. Chem. Soc. 130, 5018–5019 (2008). 18361492 10.1021/ja711023z

[b14] Iwabuchi A., Choo C.-K. & Tanaka K. Titania Nanoparticles Prepared with Pulsed Laser Ablation of Rutile Single Crystals in Water. J. Phys. B: At., Mol. Opt. Phys. 108, 10863–10871 (2004).

[b15] Wesolowski D. J. *et al.* Comment on “structure and dynamics of liquid water on rutile TiO_2_(110)”. Phys. Rev. B 85, 167401 (2012).

[b16] Liu L.-M., Zhang C., Thornton G. & Michaelides A. Structure and dynamics of liquid water on rutile TiO_2_(110). Phys. Rev. B 82, 161415 (2010).

[b17] Bolis V. *et al.* Hydrophilic/hydrophobic features of TiO_2_ nanoparticles as a function of crystal phase, surface area and coating, in relation to their potential toxicity in peripheral nervous system. J. Colloid Interface Sci. 369, 28–39 (2012). 22209580 10.1016/j.jcis.2011.11.058

[b18] Mysak E. R., Starr D. E., Wilson K. R. & Bluhm H. Note: A Combined Aerodynamic lens/ambient Pressure x-ray Photoelectron Spectroscopy Experiment for the On-stream Investigation of Aerosol Surfaces. Rev. Sci. Instrum. 81, 016106 (2010). 20113137 10.1063/1.3276714

[b19] Meinen J. *et al.* Core level Photoionization on Free sub-10-nm Nanoparticles using Synchrotron Radiation. Rev. Sci. Instrum. 81, 085107 (2010). 20815628 10.1063/1.3475154

[b20] Sublemontier O. *et al.* X-ray Photoelectron Spectroscopy of Isolated Nanoparticles. J. Phys. Chem. Lett. 5, 3399–3403 (2014). 26278452 10.1021/jz501532c

[b21] Spurr R. A. & Myers H. Quantitative Analysis of Anatase-Rutile Mixtures with an X-Ray Diffractometer. Anal. Chem. 29, 760–762 (1957).

[b22] Kavathekar R. S., English N. J. & MacElroy J. Spatial Distribution of Adsorbed Water Layers at the TiO_2_ Rutile and Anatase Interfaces. Chem. Phys. Lett. 554, 102–106 (2012).

[b23] Vittadini A., Selloni A., Rotzinger F. P. & Grätzel M. Structure and Energetics of Water Adsorbed at TiO_2_ Anatase (101) and (001) Surfaces. Phys. Rev. Lett. 81, 3–6 (1998).

[b24] Henderson M. A. Structural Sensitivity in the Dissociation of Water on TiO_2_ Single-Crystal Surfaces. Langmuir 7463, 5093–5098 (1996).

[b25] Rath C., Mohanty P., Pandey A. C. & Mishra N. C. Oxygen Vacancy induced Structural Phase Transformation in TiO_2_ Nanoparticles. J. Phys. D: Appl. Phys. 42, 205101 (2009).

[b26] Yeh S.-W. *et al.* Characteristics and Properties of a Novel *in situ* method of Synthesizing Mesoporous TiO_2_ Nanopowders by a simple Coprecipitation Process without adding Surfactant. J. Alloys Compd. 613, 107–116 (2014).

[b27] Seah M. & Dench W. Quantitative Electron Spectroscopy of Surfaces: A standard Data Base for Electron Inelastic Mean Free Path in Solids. Surf. Interface Anal. 1, 2–11 (1979).

[b28] Mårtensson N. & Nilsson A. Core-Level line shapes of adsorbates: effects of electronic and vibrational excitations. J. Electron Spectrosc. Rel. Phen. 52, 1–46 (1990).

[b29] Bancroft G. M. *et al.* Toward a comprehensive understanding of solid-state core-level XPS linewidths: Experimental and theoretical studies on the Si 2p and O 1s linewidths in silicates. Phys. Rev. B 80, 075405 (2009).

[b30] Primet M., Pichat P. & Mathieu M. V. Infrared study of the surface of titanium dioxides. I. Hydroxyl groups. J. Phys. Chem. 75, 1216–1220 (1971).

[b31] Matharu J., Cabailh G. & Thornton G. Synthesis of TiO_2_(110) ultra-thin films on W(100) and their reactions with H_2_O. Surf. Sci. 616, 198–205 (2013).

[b32] Perron H. *et al.* Combined investigation of water sorption on TiO_2_ rutile (110) single crystal face: XPS vs. periodic DFT. Surf. Sci. 601, 518–527 (2007).

[b33] Wang L.-Q., Baer D., Engelhard M. & Shultz A. The adsorption of liquid and vapor water on TiO_2_(110) surfaces: the role of defects. Surf. Sci. 344, 237–250 (1995).

[b34] Södergren S., Siegbahn H., Lindstro H., Hagfeldt A. & Lindquist S.-E. Lithium Intercalation in Nanoporous Anatase TiO_2_ Studied with XPS. J. Phys. Chem. B 101, 3087–3090 (1997).

[b35] Bullock E. L., Patthey L. & Steinemann S. G. Clean and hydroxylated rutile TiO_2_ (110) surfaces studied by x-ray photoelectron spectroscopy. Surf. Sci. 352–354, 504–510 (1996).

[b36] Sham T. K. & Lazarus M. S. X-Ray photoelectron spectroscopy (XPS) studies of clean and hydrated TiO_2_ (rutile) surfaces. Chem. Phys. Lett. 68, 426–432 (1979).

[b37] Walle L. E., Borg A., Uvdal P. & Sandell A. Probing the influence from residual Ti interstitials on water adsorption on TiO_2_ (110). Phys. Rev. B 86, 205415 (2012).

[b38] Jouan P., Peignon C., Cardinaud C. & Lempérière G. Characterisation of TiN coatings and of the TiN/Si interface by X-ray photoelectron spectroscopy and Auger electron spectroscopy. Appl. Surf. Sci. 68, 595–603 (1993).

[b39] Benoit R., Durand Y., Narjoux G. & Quintana G. Spectra and data base for XPS, AES, UPS and ESCA, photoelectrons. Available at: http://www.lasurface.com/database/elementxps.php (Accessed: 8th December 2014).

[b40] Hugenschmidt M. B., Gamble L. & Campbell C. T. The interaction of H_2_O with a TiO_2_(110) surface. Surf. Sci. 302, 329–340 (1994).

[b41] Walle L. E., Borg A., Uvdal P. & Sandell A. Experimental Evidence for Mixed Dissociative and Molecular Adsorption of Water on a Rutile TiO_2_(110) Surface Without Oxygen Vacancies. Phys. Rev. B 80, 235436 (2009).

[b42] Kollbek K. *et al.* X-ray Spectroscopic Methods in the Studies of Nonstoichiometric TiO_2_-x Thin Films. Appl. Surf. Sci. 281, 100–104 (2013).

[b43] Yamamoto S. *et al.* *In-situ* x-ray Photoelectron Spectroscopy Studies of Water on Metals and Oxides at Ambient Conditions. J. Phys.: Condens. Matter 20, 184025 (2007).

[b44] Henderson M. A. The interaction of water with solid surfaces: fundamental aspects revisited. Surf. Sci. Rep. 46, 1–308 (2002).

[b45] Kimura K., Katsumata S., Achiba Y., Yamazaki T. & Iwata S. Handbook of HeI photoelectron spectra of fundamental organic molecules (Japan Scientific Societies Press, 1981).

[b46] Kurtz R. L., Stockbauer R., Madey T. E., Roman E. & Segovia J. L. D. E. Synchrotron Radiation Studies of H_2_O Adsorption on TiO_2_(110). Surf. Sci. 218, 178–200 (1989).

[b47] Patel M., Mallia G., Liborio L. & Harrison N. M. Water adsorption on rutile TiO_2_(110) for applications in solar hydrogen production: A systematic hybrid-exchange density functional study. Phys. Rev. B 86, 045302 (2012).

[b48] Yim C. M., Pang C. L. & Thornton G. Oxygen Vacancy Origin of the Surface Band-Gap State of TiO_2_(110). Phys. Rev. Lett. 104, 036806 (2010). 20366672 10.1103/PhysRevLett.104.036806

[b49] Cheng H. & Selloni A. Hydroxide ions at the water/anatase TiO_2_(101) interface: structure and electronic states from first principles molecular dynamics. Langmuir 26, 11518–11525 (2010). 20481448 10.1021/la100672f

[b50] Söderström J. *et al.* Angle-resolved electron spectroscopy of the resonant Auger decay in xenon with meV energy resolution. New J. Phys. 13, 073014 (2011).

[b51] Gharaibeh M. F. *et al.* K-shell photoionization of singly ionized atomic nitrogen: experiment and theory. J. Phys. B: At., Mol. Opt. Phys. 44, 175208 (2011).

[b52] Lindblad A. *et al.* Vibrational scattering anisotropy in O_2_ dynamics beyond the Born-Oppenheimer approximation. New J. Phys. 14, 113018 (2012).

[b53] Kimberg V. *et al.* Single-molecule x-ray interferometry: Controlling coupled electron-nuclear quantum dynamics and imaging molecular potentials by ultrahigh-resolution resonant photoemission and ab initio calculations. Phys. Rev. X 3, 011017 (2013).

[b54] Travnikova O. *et al.* On Routes to Ultrafast Dissociation of Polyatomic Molecules. J. Phys. Chem. Lett. 4, 2361–2366 (2013).

[b55] Miron C. *et al.* Site-selective Photoemission from Delocalized Valence Shells induced by Molecular Rotation. Nat. Commun. 5, 3816 (2014). 24809410 10.1038/ncomms4816

[b56] Milosavljevic A. R. *et al.* Gas-phase Protein Inner-Shell Spectroscopy by coupling an Ion trap with a Soft x-ray Beamline. J. Phys. Chem. Lett. 3, 1191–1196 (2012). 26288054 10.1021/jz300324z

[b57] Milosavljevic A. R. *et al.* K-Shell Excitation and Ionization of a Gas-Phase Protein: Interplay between Electronic Structure and Protein Folding. J. Phys. Chem. Lett. **6**, 3132–3138 (2015).

[b58] Patanen M., Nicolas C., Liu X.-J., Travnikova O. & Miron C. Structural characterization of small Xe clusters using their 5s correlation satellite electron spectrum. Phys. Chem. Chem. Phys. 15, 10112–10117 (2013). 23702903 10.1039/c3cp50249j

[b59] Miron C. & Patanen M. Synchrotron-Radiation-Based Soft X-Ray Electron Spectroscopy Applied to Structural and Chemical Characterization of Isolated Species, from Molecules to Nanoparticles. Adv. Mater. 26, 7911–7916 (2014). 24902675 10.1002/adma.201304837

[b60] Zhang X. *et al.* A Numerical Characterization of Particle Beam Collimation by an Aerodynamic Lens-Nozzle System: Part I. An Individual Lens or Nozzle. Aerosol Sci. Technol. 36, 617–631 (2002).

[b61] Wang X., Kruis F. E. & McMurry P. H. Aerodynamic Focusing of Nanoparticles: I. Guidelines for Designing Aerodynamic Lenses for Nanoparticles. Aerosol Sci. Technol. 39, 611–623 (2005).

[b62] Wang X., Gidwani A., Girshick S. L. & McMurry P. H. Aerodynamic Focusing of Nanoparticles: II. Numerical Simulation of Particle Motion Through Aerodynamic Lenses. Aerosol Sci. Technol. 39, 624–636 (2005).

[b63] Wang X. & McMurry P. H. An experimental study of nanoparticle focusing with aerodynamic lenses. Int. J. Mass Spectrom. 258, 30–36 (2006).

[b64] Lindblad A., Söderström J., Nicolas C., Robert E. & Miron C. A multi purpose source chamber at the PLEIADES beamline at SOLEIL for spectroscopic studies of isolated species: Cold molecules, clusters, and nanoparticles. Rev. Sci. Instrum. 84, 113105 (2013). 24289386 10.1063/1.4829718

