# Supplementary material for: Water adsorption on TiO2 surfaces probed by soft X-ray spectroscopies: bulk materials vs. isolated nanoparticles
Source: Sci Rep. 2015 Oct 14;5:15088. doi: 10.1038/srep15088 (PMC4604456; doi:10.1038/srep15088)
Supplement: Supplementary Information [file srep15088-s1.doc]

**Water adsorption on TiO_2_ surfaces probed by soft X-ray spectroscopies: bulk materials vs. isolated nanoparticles**

Safia Benkoula^1^, Olivier Sublemontier^2^, Minna Patanen^1^, Christophe Nicolas^1^, Fausto Sirotti^1^ Ahmed Naitabdi^3^, François Gaie-Levrel^4^, Egill Antonsson^1^, Damien Aureau^5^, François-Xavier Ouf^6^, Shin-Ichi Wada^7^, Arnaud Etcheberry^5^, Kiyoshi Ueda^8^, Catalin Miron^1,9^

^1^ Synchrotron SOLEIL, L'Orme des Merisiers, Saint-Aubin, BP 48, 91192 Gif-sur-Yvette Cedex, France

^2^ CEA/IRAMIS/NIMBE/Laboratoire Edifices Nanométriques, CEA Saclay, 91191 Gif-sur-Yvette, France

^3^ Sorbonne Université UPMC, Univ Paris 6, UMR 7614, Laboratoire de Chimie Physique Matière et Rayonnement, 11 rue Pierre et Marie Curie, 75005 Paris, France

^4^ Laboratoire national de métrologie et d'essais, département "métrologie des gaz et des aérosols", 1 rue Gaston Boissier, 75724 Paris Cedex 15

^5^ Institut Lavoisier de Versailles, Université Versailles-St Quentin, UMR CNRS 8180, 78035 Versailles, France

^6^ Institut de Radioprotection et de Sûreté Nucléaire (IRSN), PSN-RES, SCA, LPMA, 91192 Gif-Sur-Yvette, France

^7^ Graduate School of Science, Hiroshima University, Higashi-Hiroshima 739-8526, Japan

^8^ IMRAM, Tohoku University, Sendai 980-8577, Japan

^9^ Extreme Light Infrastructure - Nuclear Physics (ELI-NP), "Horia Hulubei" National Institute for Physics and Nuclear Engineering, 30 Reactorului Street, RO-077125 Magurele, Jud. Ilfov, Romania

O1s XPS spectrum obtained on the as-received TiO_2_ nanopowder


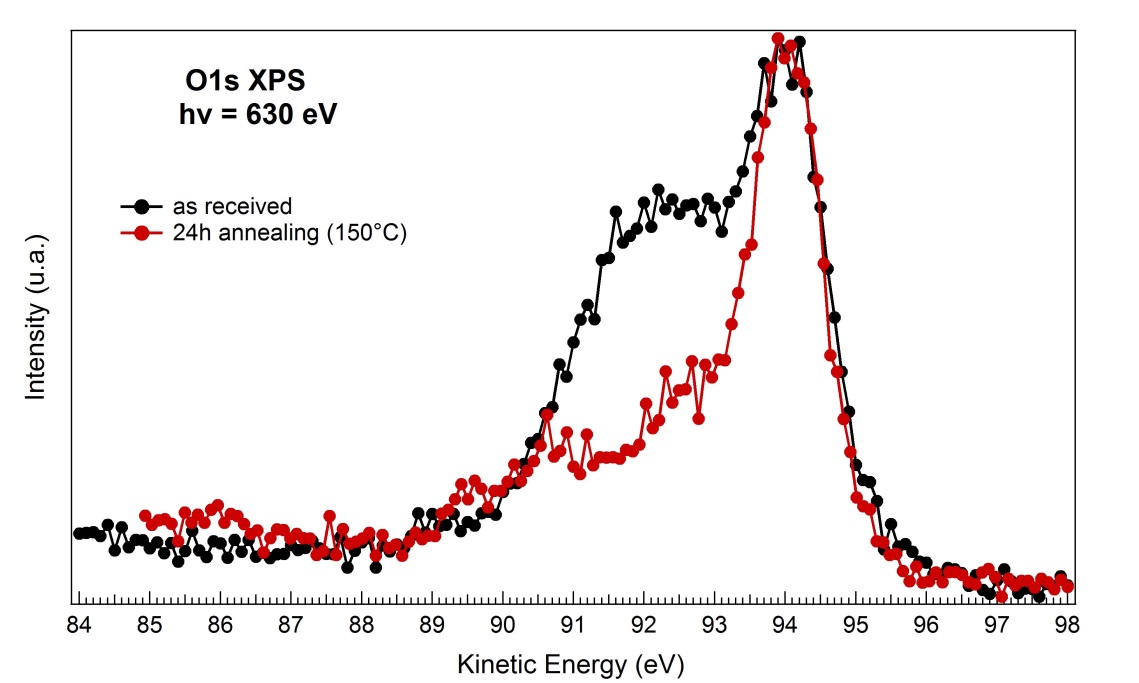


**Supplementary Figure S1:** O1s XPS spectra (in KE scale, here not calibrated in energy) recorded on as-received and on annealed TiO_2_ nanopowder (mixture of rutile and anatase). The as-received sample exhibits a double-peak shape, highlighting the hydration by ambient moisture.

Fit parameters for O1s spectra

photon energy : hv = 630 eV

experimental broadening (monochromator and electron spectrometer) = 960 meV

|  | O_bulk_ | O_br_ | H_2_O | O_contamination_ |
| --- | --- | --- | --- | --- |
| annealed TiO_2_ NP (Fig. 7a) | 1.0 | 1.1 | 1.2 | 0.8 |
| hydrated TiO_2_  (Fig. 7b) | 1.2 | 1.5 | 1.4 | 1.2 |

**Supplementary Table S2:** FWHM (in eV) of each component used to fit the O1s TiO_2_ NP spectra.

|  | O_bulk_ | O_br_ | H_2_O | O_contamination_ |
| --- | --- | --- | --- | --- |
| annealed TiO_2_ NP (Fig. 7a) | 533.9 | 535.6 | 537.4 | 538.3 |
| hydrated TiO_2_  (Fig. 7b) | 534.0 | 535.6 | 537.4 | 539.1 |

**Supplementary Table S3:** Positions (in eV +/- 0.2 eV) of each component used to fit the O1s TiO_2_ NP spectra.
